# Wikis and Collaborative Writing Applications in Health Care: A Scoping Review Protocol

**DOI:** 10.2196/resprot.1993

**Published:** 2012-04-11

**Authors:** Patrick Michel Archambault, Tom H van de Belt, Francisco J Grajales III, Gunther Eysenbach, Karine Aubin, Irving Gold, Marie-Pierre Gagnon, Craig E Kuziemsky, Alexis F Turgeon, Julien Poitras, Marjan J Faber, Jan A.M Kremer, Marcel Heldoorn, Andrea Bilodeau, France Légaré

**Affiliations:** 1 Centre de santé et de services sociaux Alphonse-Desjardins (Centre hospitalier affilié universitaire de Lévis)Lévis, QCCanada; 2 Département de médecine familiale et médecine d'urgenceUniversité LavalQuébec, QCCanada; 3 Division de soins intensifsDépartement d'anesthésiologieUniversité LavalQuébec, QCCanada; 4 Axe Traumatologie – Urgence – Soins IntensifsCentre de recherche FRQS du CHA universitaire de QuébecQuébec, QCCanada; 5 Radboud University Nijmegen Medical CentreDepartment of Obstetrics and Gynaecology, Division of Reproductive MedicineNijmegenNetherlands; 6 Radboud University Nijmegen Medical CentreRadboud REshape and Innovation CentreNijmegenNetherlands; 7 Social Media Working GroupInternational Medical Informatics AssociationGenevaSwitzerland; 8 eHealth Strategy OfficeFaculty of MedicineUniversity of British ColumbiaVancouver, BCCanada; 9 Centre for Global eHealth InnovationUniversity of Toronto and University Health NetworkToronto, ONCanada; 10 Faculté des sciences infirmièresUniversité LavalQuébec, QCCanada; 11 Association of Faculties of Medicine of CanadaOttawa, ONCanada; 12 Telfer School of ManagementUniversity of OttawaOttawa, ONCanada; 13 Radboud University Nijmegen Medical CentreScientific Institute for Quality of HealthcareNijmegenNetherlands; 14 Federation of Patients and Consumer Organisations in the NetherlandsUtrechtNetherlands; 15 Centre de recherche du Centre hospitalier universitaire de Québec (CRCHUQ)Quebec, QCCanada; 16 Canada Research Chair in Implementation of Shared Decision Making in Primary CareQuebec, QCCanada

## Abstract

The rapid rise in the use of collaborative writing applications (eg, wikis, Google Documents, and Google Knol) has created the need for a systematic synthesis of the evidence of their impact as knowledge translation (KT) tools in the health care sector and for an inventory of the factors that affect their use. While researchers have conducted systematic reviews on a range of software-based information and communication technologies as well as other social media (eg, virtual communities of practice, virtual peer-to-peer communities, and electronic support groups), none have reviewed collaborative writing applications in the medical sector. The overarching goal of this project is to explore the depth and breadth of evidence for the use of collaborative writing applications in health care. Thus, the purposes of this scoping review will be to (1) map the literature on collaborative writing applications; (2) compare the applications’ features; (3) describe the evidence of each application’s positive and negative effects as a KT intervention in health care; (4) inventory and describe the barriers and facilitators that affect the applications’ use; and (5) produce an action plan and a research agenda. A six-stage framework for scoping reviews will be used: (1) identifying the research question; (2) identifying relevant studies within the selected databases (using the EPPI-Reviewer software to classify the studies); (3) selecting studies (an iterative process in which two reviewers search the literature, refine the search strategy, and review articles for inclusion); (4) charting the data (using EPPI-Reviewer’s data-charting form); (5) collating, summarizing, and reporting the results (performing a descriptive, numerical, and interpretive synthesis); and (6) consulting knowledge users during three planned meetings. Since this scoping review concerns the use of collaborative writing applications as KT interventions in health care, we will use the Knowledge to Action (KTA) framework to describe and compare the various studies and collaborative writing projects we find. In addition to guiding the use of collaborative writing applications in health care, this scoping review will advance the science of KT by testing tools that could be used to evaluate other social media. We also expect to identify areas that require further systematic reviews and primary research and to produce a highly relevant research agenda that explores and leverages the potential of collaborative writing software. To date, this is the first study to use the KTA framework to study the role collaborative writing applications in KT, and the first to involve three national and international institutional knowledge users as part of the research process.

## Introduction

### Collaborative Writing Applications and their Potential Impact on Global Knowledge Translation

In both developed and developing countries, vast numbers of health care decision makers—providers, patients, managers, and policy makers—are failing to use research evidence to inform their decisions [1]. According to behavior change theories [2-4], self-efficacy is one of the most important cognitive determinants of behavior. By involving knowledge users in the dissemination of knowledge [5], social media—highly accessible, interactive vehicles of communication—have the potential to increase users’ self-efficacy [5-7] and empower users to apply knowledge in practice. Acknowledging this potential and recognizing that social media capitalizes on the free and open access to information, scientists, opinion leaders, and patient advocates have called for more research to determine whether social media can equip decision-making constituencies to improve the delivery of health care [8,9], decrease its cost [5,10], and improve access to knowledge within developing countries [8,11,12].

Collaborative writing applications [13,14] are a category of social media that has enjoyed a surge in popularity in recent years including within the health care sector [5,7,8,13]. Although no two applications are identical, all consist of software that allows users to create online content that anyone can edit or supplement [15]. Thus, Internet users have turned to wikis [16,17] to produce a Wikipedia entry on the Global Plan to Stop Tuberculosis [8]; to Google Knol [18] to exchange research on influenza at the Public Library of Science [19]; and to Google Docs [14,20] to review the literature on emergency medicine [21,22].

While new collaborative writing applications are continually surfacing, wikis are perhaps the most popular. Wikipedia’s medical articles are viewed about 150 million times per month and exist in 271 languages [8]. New wikis have appeared in all fields of health care [13,21,23-30], and studies of developed countries found that 70% of junior physicians use Wikipedia in any given week, that 50% to 70% of practicing physicians use it as a source of information in providing care [8,31], and that 35% of pharmacists refer to it for drug information [32]. Patients also use wikis to share their experiences [33] and to find information [8]. The Canadian Agency for Drugs and Technologies in Health (CADTH) is exploring the use of wikis to update knowledge syntheses [34,35] and the United States’ National Institutes of Health (NIH) is training its scientists in editing them [36]. In addition, academic institutions like Harvard [37] and Stanford [13] are using wikis to train health care professionals [13,16,38-43]. Wikis have come to exemplify social media’s tremendous promise to enable health care professionals, patients, and policy makers to implement evidence-based practice at remarkably low cost [21,22,44-46]. In doing so, they could improve the health of millions of people around the world [8,12].

### Knowledge Users’ Needs

Even as decision makers increase their use of wikis and other collaborative writing applications, questions remain about their safety [47,48], their reliability [49-53], their lack of traditional authorship [54,55], and the legal implications for decision making [56,57]. Researchers also question clinicians’ intention to use the applications in their practice [21] and to contribute knowledge collaboratively [8,22]. For these reasons, the International Medical Informatics Association (IMIA), the Association of Faculties of Medicine of Canada (AFMC), and the Federation of Patients and Consumer Organization in the Netherlands (NPCF) have partnered with our research team to conduct a scoping review to determine the extent of published evidence on these questions.

The Canadian Institutes of Health Research (CIHR) define a knowledge user as “an individual who is likely to be able to use the knowledge generated through research to make informed decisions about health policies, programs, and/or practices [58].” A knowledge user includes, but is not limited to, a practitioner, policy maker, educator, decision maker, health care administrator, community leader, or an individual in a health charity, patient group, private sector organization, or media outlet. In knowledge syntheses like this scoping review, CIHR requires that designated knowledge users be actively involved in all aspects. In line with this definition, the designated knowledge users in this project are IMIA, AFMC, and NPFC. These three organizations represent three different groups of stakeholders interested in the findings of this scoping review. They have been involved from the beginning of this project and will play an essential role in the dissemination and implementation of its results.

The world body for health and biomedical informatics is the IMIA [59]. As an “association of associations,” the IMIA acts as a bridge between its constituent nationally based informatics associations and its academic and industry members from around the world, and further to all interested organizations and individuals. The IMIA has a seat at the World Health Organization’s (WHO) World Health Assembly, which aims to (1) promote informatics in health care and biomedical research; (2) advance international cooperation; (3) stimulate research, development, and education in this domain; and (4) disseminate and exchange information in this domain.

Representing Canada’s 17 faculties of medicine, AFMC is the voice of academic medicine in Canada [60]. The member faculties of AFMC graduate over 2300 physicians each year; have 10,148 undergraduate medical students in training and 12,453 postgraduate trainees; and employ 21,687 full- and part-time faculty members. Thus, AFMC is a leading advocate on issues relating to health education, health research, and clinical care. Recently, AFMC has embarked on a series of projects aimed at meeting changing societal needs with innovative educational programs based on e-learning and social media. For example, in 2008, AFMC initiated the Canadian Healthcare Education Commons [61], whose mission is to provide an online environment—including wikis among other tools—to share educational material, designs, and practices in whatever form across the health care continuum and between professions in Canada.

In the Netherlands, the NPCF brings together hundreds of patient and consumer organizations to speak as one voice in areas of common interest, such as patients’ rights and access to care [62]. In the NPCF’s vision, eHealth is an essential enabler for real empowerment of patients and self-management of their health. Patient participation is very important for improving health care as the views and experiences of patients and consumers can be heard in order to shift towards a participatory health care model.

As designated knowledge users for this CIHR-funded research project, these three institutions (IMIA, AFMC, and NPCF) have helped define the need for this scoping review. In particular, these institutions want to explore the features that explain wikis and collaborative writing applications’ rising popularity [6,16] and clarify the differences between wikis and other applications, like Google Knol [8,18,19,63,64] and Google Docs [20,22]. Specifically, these institutions need to know how various applications can enhance the delivery of health care (eg, by empowering patients in decision making [65,66]), improve health care communication and education [13,20,38,67,68], and benefit health in developing countries [8]. These institutions intend to use this evidence to formulate policies for the applications’ safe and effective use.

### Gaps in the Knowledge Addressed by this Proposal

We have seen that the rapid rise in the use of collaborative writing applications in health care has created a need for a systematic synthesis of the evidence concerning their potential impacts and an inventory of the barriers and facilitators that affect their use. A scoping review is the ideal methodology to employ for a number of reasons. According to the CIHR, a scoping review is explorative and used when the relevant literature is considered to be broad and diverse as is the expanding literature about collaborative writing applications [69]. Moreover, the study of these applications is an emerging field that is being examined with diverse methods [20,38,50], with different theoretical frameworks [21], and in different contexts [35,70]. While researchers have conducted systematic reviews on information and communication technologies [71,72] and other social media (virtual communities of practice [73], virtual peer-to-peer communities, and electronic support groups [74]), none have reviewed collaborative writing applications. Therefore, in synergy and partnership with three national and international institutional knowledge users, we propose a scoping review that will map the literature on the use of wikis and other collaborative writing applications in health care in order to synthesize the applications’ positive and negative impacts and inventory the barriers and facilitators that affect how they influence the delivery of health care.

### Purposes for Conducting this Scoping Review

The overarching goal of this project is to explore the depth and breadth of evidence about the effective, safe, and ethical use of collaborative writing applications in health care systems around the world.

Specifically, the purposes of conducting this scoping review are to:

1. Map the literature on collaborative writing applications (including wikis, Google Knol, and Google Docs) in health care;

2. Compare the applications’ features by investigating how they are used in collaborative writing projects;

3. Describe the evidence of each application’s positive and negative effects as a knowledge translation (KT) intervention in health care;

4. Inventory and describe the barriers and facilitators that affect the applications’ use; and

5. Produce an action plan and a research agenda delimitating three areas: where sufficient evidence exists to make clear and judicious policy recommendations about the use of collaborative writing applications in health care, where further knowledge synthesis is needed, and where more primary research remains to be done.

### Conceptual Frameworks

Since this scoping review concerns the use of collaborative writing applications as KT interventions in health care, we will use the Knowledge to Action (KTA) framework [75,76] to describe and compare the various studies and collaborative writing projects we find. We intend to use the framework as a roadmap for determining where studies of collaborative writing applications and real projects that use those applications fit along the KT continuum. The role of collaborative writing applications in KT has not yet been determined: it is possible that applications play a different role at different phases in the KTA process. For example, a wiki used to update a systematic review [34,35] would not play the same role as a wiki used to promote global public health [8], a Google Knol used to exchange knowledge about influenza [19], or Google Docs used to teach scientific writing [20]. Finding and categorizing studies and collaborative writing projects will identify gaps in the knowledge about the applications’ use as KT interventions. These gaps will then inform our production of a research agenda.

Finally, we will describe how the studies use different behavioral and organizational models of change [79,80] to study collaborative writing applications. We will also use the taxonomy from a systematic review on the factors affecting the adoption of information and communication technology to inventory and describe the barriers and facilitators identified in this scoping review [72].

## Methods

**Figure 1:**
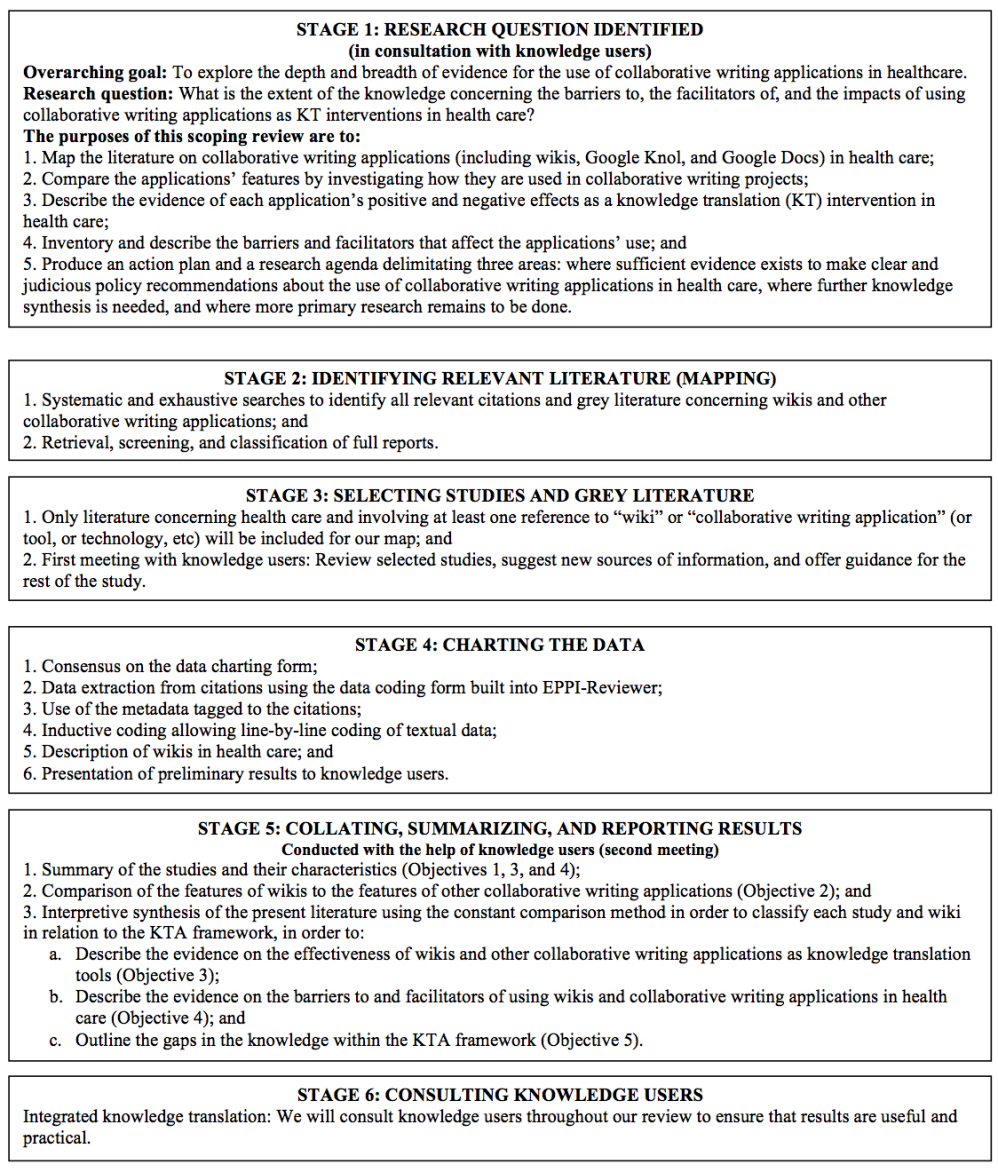
Stages of the scoping review.

To accomplish the purposes of this scoping review, we will employ the scoping review methodology described by Arksey and O’Malley [79] and further developed by Levac et al [80]. This methodology has six stages: (1) identifying the research question; (2) identifying relevant studies; (3) selecting studies; (4) charting the data; (5) collating, summarizing, and reporting the results; and (6) consulting knowledge users (Figure 1).

### Stage 1: Identifying the Research Question

The research question was developed by consulting the knowledge users to determine their needs and questions about using collaborative writing applications for KT. Their questions can be summarized as follows: “What is the extent of the knowledge concerning the barriers to, the facilitators of, and the impacts of using collaborative writing applications as KT interventions in health care?” As was previously stated, and in response to this question, the overarching goal of this project is to explore the depth and breadth of evidence about the effective, safe, and ethical use of collaborative writing applications in health care systems around the world. The purposes of our scoping review will be used to attain this goal, and therefore orient our search for publications and the grey literature. The participants targeted by this scoping review are any person involved in a KT intervention in health care (eg, patients, health care professionals, policy makers, students, educators, providers, managers, and researchers). For the purposes of our study and having referred to the writing on the subject [14-16], we have defined “collaborative writing applications” as a category of social media that enables the joint and simultaneous editing of a webpage or an online document by many end users [15]. Thus, the term covers wikis, Google Knol, and Google Docs, but does not exclude new applications for use in a future update. In terms of outcomes, our scoping review will apply no restrictions since it is important that we describe all relevant outcomes used in the literature.

### Stage 2: Identifying Studies and the Grey Literature

We will begin by comprehensively mapping publications and the grey literature to identify all sources of information within the broad remit of our overall question. To facilitate this stage, we will use software developed by the Evidence for Policy and Practice Information and Co-ordinating Centre (EPPI-Centre) [81]. Using EPPI-Reviewer 4.0 [81-85], we will create a database of publications and grey literature on collaborative writing applications in health care. EPPI-Reviewer is a multi-user web-based application for managing and analyzing data for use in research synthesis. The search methods that will be used for identifying studies and the grey literature are described below.

#### Electronic Searches

We will search publications identified in the following bibliographic databases: the Cochrane Effective Practice and Organisation of Care (EPOC) Review Group Specialised Register; the Cochrane Library (including Cochrane Database of Systematic Reviews, Cochrane Central Register of Controlled Trials, Database of Abstracts of Reviews of Effects, Health Technology Assessment Database, and NHS Economic Evaluation Database); EMBASE; PubMed; CINAHL; PsycINFO; Education Resources Information Center (ERIC); and ProQuest Dissertations and Theses. Our team’s information specialist (KA) developed a search strategy, which was peer-reviewed by an information specialist from the Medical Library of the Radboud University Nijmegen Medical Centre in The Netherlands. The search strategy is broad enough to generate an extensive map of the literature on wikis and other collaborative writing applications. We will impose no restrictions on language or date. Our preliminary search strategy (Multimedia Appendix 1), which used the terms “wiki,” “wikis,” “Web 2.0,” “social media,” “Google Knol,” “Google Docs,” and “collaborative writing applications,” identified 7174 citations before removal of duplicates.

#### Other Sources

We will conduct additional searches by (1) scanning the reference lists of included studies; (2) reviewing the two most recent editions of the proceedings and abstracts of relevant conferences, symposia, and colloquia; (3) searching web-based registries of clinical trials; (4) contacting experts to request details of any known studies (eg, the authors of WikiProject Medicine [8]); and (5) searching the following repositories of grey literature: the New York Academy of Medicine Library’s Grey Literature Report, OpenSIGLE, the Health Technology Assessment international (HTAi) Vortal, and CADTH’s online search engine.

We will also search for grey literature on the Internet using the search engines Google, Bing, Yahoo, Mednar, and Scopus. Google, Bing, and Yahoo are the most widely used search engines [85]; Mednar and Scopus focus on scientific content. We will use the advanced search option, select no preferred language, and turn off the option for regional differences. Based on previous research [85,86], we expect a large number of results. For this reason, when searching with Google, Yahoo, Bing, and Scopus, we will use a more specific search string query, such as “wiki in health care,” “Google Knol in health care,” “Google Docs in health care,” and “collaborative writing applications in health care.” We will study the first 100 results in Google, Bing, and Yahoo, which all display results by relevance using a link analysis system or algorithms [85]. We will then analyze the top 100 results for each search engine to identify all collaborative writing projects inventoried. We will complete our comprehensive search of the Internet by consulting existing lists of wikis in health care [23,87]. The founding authors of each identified collaborative writing project will be contacted and asked for all published or unpublished descriptions of the features of the application they used (eg, wiki, Google Knol, or Google Docs), studies of the impacts of the application, and studies of the barriers to, and facilitators of, the use of the application.

To ensure we include all relevant studies, we will invite all interested Internet users and researchers to share papers that could potentially fall within the scope of this review. A public online Mendeley library has been created to allow anyone to make contributions to the current collection of citations. To add citations to this online library, interested individuals are invited to access the library [88]. Furthermore, if interested individuals prefer to use a wiki to share their citations, they are invited to do so by using the HLWIKI [89]. A Google Docs spreadsheet [90] will also allow potential collaborators to add citations for consideration for this scoping review. We will use these different social media resources to verify if any new citations will be identified by comparing the lists of citations created in these three resources to the lists we will be creating within EPPI-Reviewer. Any individual’s contribution to these three resources will be recognized and appropriately credited.

#### EPPI-Reviewer

All sources of information (publications and grey literature) will be imported into EPPI-Reviewer using the Research Information Systems (RIS) tagging format. For webpages, we will use Mendeley [91], a free online reference manager built to facilitate the tagging and describing of web-based sources of information. We will then import these tagged webpages in RIS format into EPPI-Reviewer for further analysis. All duplicates will be removed within EPPI-Reviewer.

### Stage 3: Selecting Studies and the Grey Literature

This stage will consist of an iterative process in which we search the literature, refine our search strategy, and review articles for inclusion. Two reviewers will independently screen all titles, abstracts, and grey literature in EPPI-Reviewer and retain only material concerning the field of health care and involving collaborative writing applications such as wikis, Google Knol, and Google Docs. The team’s reviewers will meet at the beginning, during the middle, and at the end of the review process to discuss their selection of literature and to refine the search strategy, if needed. Two reviewers will then independently review full articles and grey literature for inclusion. If they disagree, a third reviewer will arbitrate. EPPI-Reviewer will facilitate consensus by allowing multiple users to classify studies independently before comparing their results. EPPI-Reviewer will also produce summary discrepancy reports. Its interface will facilitate final decisions.

### Stage 4: Charting the Data

We have already developed a preliminary data-charting form and determined which information to extract. This form will be built into EPPI-Reviewer to facilitate our coding of data. Two authors will use the form to extract data from the first 10 studies and/or grey literature independently before meeting to determine whether their approach to data extraction is consistent with the research question and the purpose of the review. Thus, for the first 10 sources of information, charting will be an iterative process in which researchers continually update the data-charting form. Once the reviewers reach consensus on the form, they will send it to all team members for final comments and suggestions, after which the reviewers will use it to extract data for each publication. The reviewers will compare their extraction results within EPPI-Reviewer. If they disagree, a third reviewer will determine the final version of the data extracted.

Using EPPI-Reviewer’s inductive coding function, which allows textual data to be coded line-by-line, and using the metadata already tagged to each citation in RIS format, two reviewers will qualitatively describe the sources of information with regard to the following variables: authorship, year of publication, country, status of publication (ie, published or grey literature), journal, Medical Subject Headings (MeSH) terms used, participants (patients, health care professionals, policy makers, educators, or students), study setting, study design (eg, experimental, non-experimental, or qualitative), collaborative writing application used in the intervention group, goal of the intervention (conducting reviews, developing guidelines, promoting evidence-based practice, promoting evidence-informed policy making, promoting shared decision making, or teaching health care), description of the comparison, description of the outcomes, description of the positive and negative impacts, description of barriers and facilitators, use of a behavioral or organizational theory of change to describe barriers and facilitators. For every collaborative writing project that involved the use of a collaborative writing application, we will code the following variables: website address, audience, contributors, editors, supporting organization, editorial policy, recognition of authorship, presence of publicity, number of pages, language, type of content, application used (eg, wiki software), references to published descriptions, references to studies assessing the project’s impact, and references to studies on barriers and facilitators. Using EPPI-Reviewer, we will compare the reviewers’ coding to ensure that our results are trustworthy. Any discrepancy will be resolved by discussion. If consensus is not possible, a third reviewer will decide.

### Stage 5: Collating, Summarizing, and Reporting Results

#### Collating and Summarizing

As described in the framework by Arksey and O’Malley [79], our analysis (referred to as “collating and summarizing”) will involve a descriptive numerical summary and an interpretive synthesis.

First, we will summarize the studies and their characteristics as described in the charting stage (Purposes 1, 3, and 4). This description will constitute our map of the literature on collaborative writing applications in health care. We will report the frequency of studies according to variables defined in Stage 4, such as the study design, the type of intervention that took place, the outcomes that were measured (health care process outcomes or health outcomes), the positive and negative impacts, the barriers and facilitators, and the explicit use (or non-use) of a theoretical framework.

Our description of impacts (Purpose 3) will remain qualitative and will serve to identify the potential for future systematic reviews. Examples of impacts are an increase in professionalism by medical students (a positive impact) [38] and the dissemination of inaccurate information on HIV/AIDS medication (a negative impact) [50]. We will begin our description by developing a coding scheme using qualitative content analysis, a method whereby researchers interpret textual data subjectively by systematically classifying and coding data and identifying patterns [92]. Using a random sample of 10% of all data, two reviewers will identify the positive and negative impacts mentioned by the studies and mark recurrent impacts with codes [92]. They will begin by reading the data repeatedly to immerse themselves and obtain a broad perspective [93]. Then, with EPPI-Reviewer’s full text mining capacity, they will read the content word-by-word, highlighting words that appear to capture impacts and assigning them codes, which they will then organize into categories. They will also develop a tree diagram to organize the categories into a hierarchical structure [94]. Next, we will develop definitions for each code and category. These codes and categories will constitute our coding scheme and will guide reviewers’ content analysis of the rest of the data. The two reviewers will discuss units of text that could not be coded and will create new codes as necessary.

Our description of barriers and facilitators (Purpose 4) will be based on a validated taxonomy developed by Gagnon et al [72]. The reviewers will read each publication independently and identify the unit of text (a sentence or paragraph representing an idea) relevant to each main outcome of interest (barriers and facilitators). Using EPPI-Reviewer, they will then code each unit of text according to the code list. If necessary, the reviewers will create new codes for units of text that cannot otherwise be coded, thus refining and expanding the list. The reviewers will resolve any coding discrepancies through discussion. During the coding process, codes will be aggregated into themes, which will be nested under a main theme.

The same constant comparison method [92] will be used to compare the features of the collaborative writing applications by analyzing their use in different collaborative writing projects (Purpose 2). Again, a coding scheme will be developed from a random sample of 10% of the data, following the process used for coding impacts. In this case, the categories will correspond to meaningful clusters that reflect the relationships between the applications’ features. We will code the data using this scheme, as per the process described previously. We will also construct a table that compares the collaborative writing applications used for each project and identifies the presence or absence of features using the developed coding scheme. The resulting synthesis will allow knowledge users—IMIA, AFMC, and NPCF in particular—to make recommendations for the use of the applications that more accurately reflect the applications’ strengths and weaknesses.

Also using the constant comparison method, we will perform directed content analysis [92] to classify each project that used a collaborative writing application in relation to the KTA framework. The KTA framework will serve as a map on which collaborative projects will be plotted according to each project’s explicit or implicit goal as interpreted by the reviewers’ analysis of the project’s features and characteristics. Thus, each project will occupy a space within the KTA framework that reflects the phase of the KTA framework that the project is likely to influence. The KTA framework will describe the phases and detail the relationships between them, helping to determine the initial coding scheme. Projects that cannot be coded will be identified and analyzed later to determine whether they represent a new process within the KTA framework or a subcategory of an existing process. This directed approach to content analysis will allow us to validate the KTA framework for the study of future collaborative writing projects. It will also allow the KTA framework to be extended if new processes or subprocesses are identified.

The conceptual framework generated by our directed content analysis will allow us to classify applications according to the phase of the KTA process that they influence. It will do likewise for applications’ positive and negative impacts (Purpose 3) and the barriers to, and facilitators of, using the applications as KT tools in health care (Purpose 4). In addition, the analysis will guide: (1) our formulation of clear, evidence-based policies where sufficient evidence exists about the use of wikis and other collaborative writing applications as KT interventions; (2) our analysis of gaps in the knowledge; and (3) our identification of areas where more primary research is needed and areas where there is enough data to conduct systematic reviews (Purpose 5).

#### Reporting Results

To present the results of our qualitative analyses, we will employ descriptive tables, frequency tables, and diagrams. A table will describe the characteristics of each study included in our review. Additional tables will classify the studies according to their principal characteristics: participants, study setting, study design, study intervention, aim of the collaborative writing applications, and outcomes studied. A summary table will group those studies that assessed the impacts of the use of a collaborative writing application, showing the phase of the KTA process that the application influenced and describing the studies’ results. Another summary table will present all the studies that assessed barriers and facilitators, the theory used by each, the KTA process influenced, and—using a validated taxonomy—a description of the barriers and facilitators found. These tables will be useful for knowledge users interested in the impacts of using collaborative writing applications in health care and on the barriers and facilitators that affect their use. To compare applications, a Venn diagram will be constructed that situates each application in relation to the others. This will help knowledge users understand how each application can be used. Finally, a diagram that situates the different collaborative writing applications within the KTA framework will help knowledge users understand the applications’ role in KT. This conceptual map will be very useful in designing systematic reviews and primary studies in the future.

### Stage 6: Consulting Knowledge Users

Our scoping review will involve the knowledge users throughout the review’s duration in order to generate usable and practical results. This integrated KT model is important to giving the review perspective, meaning, applicability, and a clear purpose. By laying out their needs for the products of this review, knowledge users have already shaped our research purposes. We will continue to involve knowledge users by conducting two teleconferences during the course of the review. In the first teleconference (after Stage 3), we will share the preliminary findings of the review to validate our findings and guide the review’s completion. This meeting will be an opportunity for IMIA, AFMC, and NPCF to identify additional sources of information that we should consider. The second, and final, meeting will be held near the end of Stage 5, when we will use the preliminary findings from Stage 5 (presented in tables and diagrams) as a foundation for the formulation of an action plan and a research agenda (Purpose 5). Our knowledge users will have the opportunity to build on the evidence presented and offer more meaning, content expertise, and perspective to the preliminary findings. These meetings will guide our writing of the final report and the two-page policy briefs that knowledge users find accessible and useful.

## Discussion

This review will generate results that will be highly pertinent to the knowledge users who will collaborate on the project, as well as to the broader community they represent. In general, it will draw upon the evidence to refine the community’s understanding of the use of collaborative writing applications as KT instruments. First, it will identify the features that differentiate collaborative writing applications; second, it will discuss the positive and negative impacts of different collaborative writing applications and the barriers and facilitators that affect their use. Using the KTA framework, we will group the applications by KTA phase. This will allow us to produce a strategic action plan that is grounded in knowledge users’ feedback and makes recommendations about the use of collaborative writing applications as KT interventions where justified by the evidence. Also, it will allow us to develop a research agenda that can identify areas that need more systematic review or primary research. Ultimately, we expect our findings to benefit knowledge users in health care organizations around the world, especially in developing countries where clinicians are most likely to value applications that share free, reliable, health information. The review will also help build a strong partnership between knowledge users and scientists, which will be useful for further research. Furthermore, knowledge users and researchers around the world are invited to pursue this endeavor in collaboration with us by contributing to the synthesis of new knowledge on wikis and collaborative writing applications in health care. This novel use of crowdsourcing to identify citations and to update the database of citations created with this study will add to the results of ongoing studies concerning the potential use of crowdsourcing to supplement the process of knowledge synthesis and scoping reviews [95,96]. In addition to contributing to the guidance on the use of collaborative writing applications, this scoping review will advance the science of KT by testing and improving tools that could be used to evaluate other social media. In particular, this review will be the first to use the KTA framework to study the role of collaborative writing applications in KT. Using this framework will help us determine a research agenda that will be instrumental in future explorations of applications such as wikis, Google Knol, and Google Docs.

### Conclusions

For all the promise and power of collaborative writing applications for KT, the applications are also fraught with important barriers and the potential of adverse effects. This argues for rapid guidelines for the implementation and development of these new social media. To date, this is the first study that will use the KTA framework to examine the role collaborative writing applications can play in KT. It is also the first to involve three national and international institutional knowledge users—IMIA, AFMC, and NPCF—in the process.

## Multimedia Appendix 1

Definitive search term strategy in different databases and number (n) of citations found for each database (October 2011).

## Abbreviations

AFMC: Association of Faculties of Medicine of Canada
CADTH: Canadian Agency for Drugs and Technologies in Health
CIHR: Canadian Institutes of Health Research
EPOC: Effective Practice and Organisation of Care
ERIC: Education Resources Information Center
IMIA: International Medical Informatics Association
KT: knowledge translation
KTA: Knowledge to Action
MeSH: Medical Subject Headings
NIH: National Institutes of Health
RIS: Research Information Systems
WHO: World Health Organization

## References

[1] Straus SE, Tetroe J, Graham ID (2009). Knowledge Translation in Health Care : Moving from Evidence to Practice.

[2] Michie S, Johnston M, Abraham C, Lawton R, Parker D, Walker A (2005). Making psychological theory useful for implementing evidence based practice: a consensus approach. Qual Saf Health Care.

[3] Godin G, Bélanger-Gravel A, Eccles M, Grimshaw J (2008). Healthcare professionals' intentions and behaviours: a systematic review of studies based on social cognitive theories. Implement Sci.

[4] Bandura A (2004). Health promotion by social cognitive means. Health Educ Behav.

[5] Eysenbach G (2008). Medicine 2.0: social networking, collaboration, participation, apomediation, and openness. J Med Internet Res.

[6] Tapscott D, Williams AD (2008). Wikinomics: How Mass Collaboration Changes Everything.

[7] McLean R, Richards BH, Wardman JI (2007). The effect of Web 2.0 on the future of medical practice and education: Darwikinian evolution or folksonomic revolution?. Med J Aust.

[8] Heilman JM, Kemmann E, Bonert M, Chatterjee A, Ragar B, Beards GM, Iberri DJ, Harvey M, Thomas B, Stomp W, Martone MF, Lodge DJ, Vondracek A, de Wolff JF, Liber C, Grover SC, Vickers TJ, Meskó B, Laurent MR (2011). Wikipedia: a key tool for global public health promotion. J Med Internet Res.

[9] Czarnecka-Kujawa K, Abdalian R, Grover SC (2008). M1042 The quality of open access and open source Internet material in gastroenterology: Is Wikipedia appropriate for knowledge transfer to patients?. Gastroenterology.

[10] Mandl KD, Kohane IS (2008). Tectonic shifts in the health information economy. N Engl J Med.

[11] de Silva V, Hanwella R (2010). Why are we copyrighting science?. BMJ.

[12] Godlee F, Pakenham-Walsh N, Ncayiyana D, Cohen B, Packer A (2004). Can we achieve health information for all by 2015?. Lancet.

[13] Chu LF, Young C, Zamora A, Kurup V, Macario A (2010). Anesthesia 2.0: internet-based information resources and Web 2.0 applications in anesthesia education. Curr Opin Anaesthesiol.

[14] Eapen BR (2007). Collaborative writing: Tools and tips. Indian J Dermatol Venereol Leprol.

[15] Kaplan AM, Haenlein M (2010). Users of the world, unite! The challenges and opportunities of Social Media. Business Horizons.

[16] Boulos MN, Maramba I, Wheeler S (2006). Wikis, blogs and podcasts: a new generation of Web-based tools for virtual collaborative clinical practice and education. BMC Med Educ.

[17] Leuf B, Cunningham W (2001). The Wiki way: quick collaboration on the Web.

[18] Levy, S Wired.

[19] Google Knol.

[20] Phadtare A, Bahmani A, Shah A, Pietrobon R (2009). Scientific writing: a randomized controlled trial comparing standard and on-line instruction. BMC Med Educ.

[21] Archambault PM, Légaré F, Lavoie A, Gagnon MP, Lapointe J, St-Jacques S, Poitras J, Aubin K, Croteau S, Pham-Dinh M (2010). Healthcare professionals' intentions to use wiki-based reminders to promote best practices in trauma care: a survey protocol. Implement Sci.

[22] Archambault PM, Blouin D, Poitras J, Couture M, Légaré F (2010). Resident participation in an internet-based collaborative teaching tool (Google Docs) [abstract 214]. Royal College Abstracts for the ICRE 2010.

[23] Rothman D (2009). davidrothman.net.

[24] Medpedia.

[25] Croteau S.

[26] Hoffmann R (2008). A wiki for the life sciences where authorship matters. Nat Genet.

[27] Wikigenes: collaborative publishing.

[28] Waldrop MM (2008). Science 2.0. Sci Am.

[29] OpenWetWare Main Page.

[30] de Carvalho EC, Batilana AP, Simkins J, Martins H, Shah J, Rajgor D, Shah A, Rockart S, Pietrobon R (2010). Application description and policy model in collaborative environment for sharing of information on epidemiological and clinical research data sets. PLoS One.

[31] Hughes B, Joshi I, Lemonde H, Wareham J (2009). Junior physician's use of Web 2.0 for information seeking and medical education: a qualitative study. Int J Med Inform.

[32] Brokowski L, Sheehan AH (2009). Evaluation of pharmacist use and perception of Wikipedia as a drug information resource. Ann Pharmacother.

[33] Wikia.

[34] Deshpande A, Khoja S, Lorca J, McKibbon A, Rizo C, Husereau D, Jadad AR (2009). Asynchronous telehealth: a scoping review of analytic studies. Open Med.

[35] McIntosh B, Cameron C, Singh S, Yu C, Ahuja T, Welton NJ, Dahl M (2011). Open Medicine Live Wiki.

[36] Caputo, I (2009). The Washington Post.

[37] Kim JY, Gudewicz TM, Dighe AS, Gilbertson JR (2010). The pathology informatics curriculum wiki: Harnessing the power of user-generated content. J Pathol Inform.

[38] Varga-Atkins T, Dangerfield P, Brigden D (2010). Developing professionalism through the use of wikis: A study with first-year undergraduate medical students. Med Teach.

[39] Kohli MD, Bradshaw JK (2011). What is a wiki, and how can it be used in resident education?. J Digit Imaging.

[40] Medical Education.

[41] Sandars J, Haythornthwaite C (2007). New horizons for e-learning in medical education: ecological and Web 2.0 perspectives. Med Teach.

[42] Sandars J, Homer M, Pell G, Crocker T (2010). Web 2.0 and social software: the medical student way of e-learning. Med Teach.

[43] McGee JB, Begg M (2008). What medical educators need to know about "Web 2.0". Med Teach.

[44] Schreiber WE, Giustini DM (2009). Pathology in the era of Web 2.0. Am J Clin Pathol.

[45] Barsky E, Giustini D (2008). Web 2.0 in physical therapy: a practical overview. Physiother Can.

[46] Giustini D (2006). How Web 2.0 is changing medicine. BMJ.

[47] Goodman MJ (2006). Readers' and author's responses to "are traditional peer-reviewed medical articles obsolete?". MedGenMed.

[48] Devgan L, Powe N, Blakey B, Makary M (2007). Wiki-Surgery? Internal validity of Wikipedia as a medical and surgical reference. Journal of the American College of Surgeons.

[49] Rosenzweig R (2006). Can History Be Open Source? Wikipedia and the Future of the Past. Journal of American History.

[50] Clauson KA, Polen HH, Boulos MN, Dzenowagis JH (2008). Scope, completeness, and accuracy of drug information in Wikipedia. Ann Pharmacother.

[51] Callis KL, Christ LR, Resasco J, Armitage DW, Ash JD, Caughlin TT, Clemmensen SF, Copeland SM, Fullman TJ, Lynch RL, Olson C, Pruner RA, Vieira-Neto EH, West-Singh R, Bruna EM (2009). Improving Wikipedia: educational opportunity and professional responsibility. Trends Ecol Evol.

[52] Pender MP, Lasserre KE, Del Mar C, Kruesi L, Anuradha S (2009). Is Wikipedia unsuitable as a clinical information resource for medical students?. Med Teach.

[53] Arita M (2009). A pitfall of wiki solution for biological databases. Brief Bioinform.

[54] Giles J (2006). Wikipedia rival calls in the experts. Nature.

[55] Kittur A, Suh B, Pendleton BA, Chi EH (2007). He says, she says: conflict and coordination in Wikipedia. Proceedings of the SIGCHI conference on Human factors in computing systems.

[56] Jain SH (2009). Practicing medicine in the age of Facebook. N Engl J Med.

[57] Cohen, N (2009). The New York Times.

[58] Canadian Institutes of Health Research.

[59] International Medical Informatics Association.

[60] The Association of Faculties of Medicine of Canada.

[61] Canadian Healthcare Education Commons - La collaboration pour l'éducation en santé au Canada.

[62] The Federation of Patients and Consumer Organisations in the Netherlands.

[63] Manber U (2007). Official Google blog.

[64] Google Knol.

[65] Adams SA (2010). Revisiting the online health information reliability debate in the wake of "web 2.0": an inter-disciplinary literature and website review. Int J Med Inform.

[66] Vogel L (2011). Dr. YouTube will see you now. CMAJ.

[67] Collier J (2010). Wiki technology in the classroom: building collaboration skills. J Nurs Educ.

[68] Naik AD, Singh H (2010). Electronic health records to coordinate decision making for complex patients: what can we learn from wiki?. Med Decis Making.

[69] ResearchNet - Canadian Institutes of Health Research.

[70] Anderson PF, Blumenthal J, Bruell D, Rosenzweig D, Conte M, Song J (2009). An online and social media training curricula to facilitate bench-to-bedside information transfer. Positioning the Profession: the Tenth International Congress on Medical Librarianship.

[71] Black AD, Car J, Pagliari C, Anandan C, Cresswell K, Bokun T, McKinstry B, Procter R, Majeed A, Sheikh A (2011). The impact of eHealth on the quality and safety of health care: a systematic overview. PLoS Med.

[72] Gagnon MP, Desmartis M, Labrecque M, Car J, Pagliari C, Pluye P, Frémont P, Gagnon J, Tremblay N, Légaré F (2012). Systematic review of factors influencing the adoption of information and communication technologies by healthcare professionals. J Med Syst.

[73] Li LC, Grimshaw JM, Nielsen C, Judd M, Coyte PC, Graham ID (2009). Use of communities of practice in business and health care sectors: a systematic review. Implement Sci.

[74] Eysenbach G, Powell J, Englesakis M, Rizo C, Stern A (2004). Health related virtual communities and electronic support groups: systematic review of the effects of online peer to peer interactions. BMJ.

[75] Graham ID, Logan J, Harrison MB, Straus SE, Tetroe J, Caswell W, Robinson N (2006). Lost in knowledge translation: time for a map?. J Contin Educ Health Prof.

[76] Graham, I (2007). Canadian Institutes of Health Research.

[77] Eccles M, Grimshaw J, Walker A, Johnston M, Pitts N (2005). Changing the behavior of healthcare professionals: the use of theory in promoting the uptake of research findings. J Clin Epidemiol.

[78] Cabana MD, Rand CS, Powe NR, Wu AW, Wilson MH, Abboud PA, Rubin HR (1999). Why don't physicians follow clinical practice guidelines? A framework for improvement. JAMA.

[79] Arksey H, O'Malley L (2005). Scoping studies: towards a methodological framework. International Journal of Social Research Methodology.

[80] Levac D, Colquhoun H, O'Brien KK (2010). Scoping studies: advancing the methodology. Implement Sci.

[81] Elbourne D, Oakley A, Gough D (2001). Collaboration with the Campbell collaboration. EPPI centre reviews will aim to disseminate systematic reviews in education. BMJ.

[82] Pope C, Mays N, Mays N, Popay J (2007). Synthesizing qualitative and quantitative health evidence: a guide to methods.

[83] Harden A, Oakley A, Oliver S (2001). Peer-delivered health promotion for young people: a systematic review of different study designs. Health Education Journal.

[84] Oliver S, Harden A, Rees R, Shepherd J, Brunton G, Garcia J, Oakley, A (2005). An emerging framework for including different types of evidence in systematic reviews for public policy. Evaluation.

[85] Van De Belt TH, Engelen LJ, Berben SA, Schoonhoven L (2010). Definition of Health 2.0 and Medicine 2.0: a systematic review. J Med Internet Res.

[86] Hughes B, Joshi I, Wareham J (2008). Health 2.0 and Medicine 2.0: tensions and controversies in the field. J Med Internet Res.

[87] HLWIKI.

[88] Mendeley.

[89] HLWIKI.

[90] Google Documents.

[91] Hull D, Pettifer SR, Kell DB (2008). Defrosting the digital library: bibliographic tools for the next generation web. PLoS Comput Biol.

[92] Hsieh HF, Shannon SE (2005). Three approaches to qualitative content analysis. Qual Health Res.

[93] Tesch R (1990). Qualitative Research; Analysis Types and Software Tools.

[94] Morse JM, Field PA (1995). Qualitative research methods for health professionals.

[95] Khoja S, Lorca J, McKibbon A, Rizo C, Husereau D, Jadad AR, Deshpande A Open Medicine wiki.

[96] Cameron C, Singh SR, Yu C, Ahuja T, Welon NJ, Dahl M, McIntosh B Open Medicine Live Wiki.

