# Supplementary material for: Wikis and Collaborative Writing Applications in Health Care: A Scoping Review Protocol
Source: JMIR Res Protoc. 2012 Apr 11;1(1):e1. doi: 10.2196/resprot.1993 (PMC3626140; doi:10.2196/resprot.1993)
Supplement: Supplementary file 1 [file resprot_v1i1e1_app1.pdf]

Multimedia appendix 1

Definitive search term strategy in different databases and number (n) of citations found for each database (October 2011)

1- **Pubmed (n=1061)**: Wiki*[All Fields] OR "Web 2.0"[TIAB] OR "Web2.0"[TIAB] OR (google* AND knol) OR (google* AND docs) OR "Social media"[TIAB] OR (Collaborative [tiab] AND writing [tiab]) OR (collaborative technolog*) OR (collaborative software*)
2- **Embase (n=1059):** wiki* OR 'collaborative technology' OR 'collaborative technologies' OR 'collaborative writing' OR 'collaborative writings' OR 'collaborative software' OR 'collaborative softwares' OR 'google docs' OR 'google knol' OR 'ehealth 2.0' OR 'health 2.0' OR ‘e+health 2.0’ OR 'web 2.0'
3- **CINAHL (n=1462)**: TI ((wiki* or "google docs" or "google knol" or "medecine 2.0." or "web 2.0" or "collaborative technolog*" or "collaborative writing" or "ehealth" or "e-health" or emedicine or "e-medicine")) OR AB ((wiki* or "google docs" or "google knol" or "medecine 2.0." or "web 2.0" or "collaborative technolog*" or "collaborative writing" or "ehealth" or "e-health" or emedicine or "e-medicine"))
4- **PsychINFO (n=1124):** (wiki* or "google docs" or "google knol" or "collaborative software" or "collaborative writing" or "collaborative technologies" or "collaborative techonology"):Any Field OR ("medicine 2.0" or "emedicine" or e-medicine or "health 2.0" or "ehealth" or e-health or "web 2.0"):Title OR ("medicine 2.0" or "emedicine" or e-medicine or "health 2.0" or "ehealth" or e-health or "web 2.0"):Abstract
5- **ERIC (n=1780):** ((Keywords:wiki* or Keywords:"web 2.0" or Keywords:"google docs" or Keywords:"google knol" or Keywords:"collaborative technologies" or Keywords:"collaborative technology" or Keywords:"collaborative software" or Keywords:"collaborative writing" or Keywords:"e-health" or Keywords:ehealth) or (Title:wiki* or Title:"web 2.0" or Title:"google docs" or Title:"google knol" or Title:"collaborative technologies" or Title:"collaborative technology" or Title:"collaborative software" or Title:"collaborative writing" or Title:"e-health" or Title:ehealth) and (Thesaurus Descriptors:"Health services"))
6- **Dissertation abstract & Thesis (n=632):** Citation & Abstract (wiki* or "health 2.0" or "web 2.0" or "e-medicine" or emedicine or "google docs" or "google knol" or "collaborative technologies" or "collaborative technology" or "collaborative writing" or "collaborative software")
7- **Cochrane Library (n=56):** (wiki* or "web 2.0" or ehealth or "e-health" or "google docs" or "google knol" or "collaborative writing") in Title, Abstract or Keywords in All Cochrane Library

TOTAL NUMBER OF CITATIONS: 7174 (no duplicates removed)
